# Neuroendocrine Carcinoma as a Cause of Humoral Hypercalcemia of Malignancy: A Case of a Patient With Elevated Parathyroid Hormone-Related Protein

**DOI:** 10.7759/cureus.23398

**Published:** 2022-03-22

**Authors:** Bryce R Christensen, Matthew J Rendo, Bradley W Beeler, Brent J Huddleston, Joshua L Fenderson

**Affiliations:** 1 Internal Medicine, Brooke Army Medical Center, San Antonio, USA; 2 Hematology and Oncology, Brooke Army Medical Center, San Antonio, USA; 3 Pathology, Brooke Army Medical Center, San Antonio, USA

**Keywords:** 1'25-dihydroxyvitamin d, parathyroid hormone-related peptide, cinacalcet, neuroendocrine tumor, humoral hypercalcemia of malignancy

## Abstract

Humoral hypercalcemia of malignancy (HHM) is a paraneoplastic syndrome caused by elevations in parathyroid hormone-related protein (PTH-rP). HHM often presents in patients with squamous cell carcinomas of the lung, head, and neck, as well as breast, ovarian, renal, and bladder carcinomas. HHM associated with neuroendocrine carcinoma (NEC) is rarely observed. Here, we report a case of NEC-associated HHM refractory to standard calcium-reducing therapies but improved with the off-label addition of cinacalcet. A 31-year-old male with metastatic NEC presented to the emergency department (ED) with symptoms of nausea, emesis, constipation, and progressive weakness. He was being treated via a clinical trial at a tertiary referral center after failing standard therapies. He had recently been admitted at an outside facility for hypercalcemia, which had been managed with denosumab (120 mg subcutaneously) over the previous four weeks. He was admitted from the ED with a serum calcium of 14.6 mg/dL, potassium of 2.9 mmol/L, and phosphate of 1.2 mg/dL; ionized calcium was elevated at 8.0 mg/dL. Despite hydration and aggressive electrolyte replacement, his calcium increased to 15.5 mg/dL. Further laboratory evaluation revealed parathyroid hormone (PTH) of 6 pg/mL (10-65 pg/mL), 25-hydroxyvitamin D of 25 ng/mL (25-80 ng/mL), 1,25-dihydroxyvitamin D of 513 pg/mL (18-64 pg/mL), and PTH-rP of 25 pmol/L (<2.5 pmol/L), consistent with HHM. Calcitonin was avoided due to a prior hypersensitivity reaction. He received prednisone 10 mg daily and pamidronate 90 mg IV, and his calcium improved to 11.5 mg/dL. He was discharged and investigational therapy was resumed. This therapy failed, and he did not qualify for additional cancer therapy due to refractory hypercalcemia. He was started on cinacalcet, and his calcium decreased enough to permit further cancer treatment. He had multiple hospitalizations with fluctuating calcium levels and ultimately died several months later after sustaining a subarachnoid hemorrhage from a fall. In conclusion, we report a rare case of HHM associated with NEC. While many cases of HHM are effectively managed with hydration, calcitonin, antiresorptive therapies, and glucocorticoids, some are refractory. Our patient was refractory and differed from most patients with HHM in at least two ways. As mentioned previously, NEC causing HHM is quite uncommon (~2% of cases); it is unclear, but this malignancy might predispose to refractory hypercalcemia. Our patient’s elevated vitamin D may also have made his HHM more resistant to treatment. Ultimately, while not first line, cinacalcet was an effective treatment in our patient. This provides additional evidence that cinacalcet may be considered for refractory hypercalcemia secondary to malignancy.

## Introduction

Humoral hypercalcemia of malignancy (HHM) is a known phenomenon in some patients with nonmetastatic solid tumors and hematological malignancies [1,2]. The most common pathology associated with HHM is squamous cell carcinoma, with the most frequent variant being squamous cell carcinoma of the lung [2]. HHM is caused by parathyroid hormone-related protein (PTH-rP), a gene product often expressed in normal keratinocytes [3]. This protein is also produced in low levels by some neuroendocrine, epithelial, and mesoderm-derived tissues and in minimal levels by essentially all tissues. PTH-rP and HHM are particularly relevant in the setting of hypercalcemia of malignancy, accounting for at least 80% of cases, with some studies attributing it to nearly 90% to 100% of cases [4-6]. PTH-rP has also been shown to cause HHM in the setting of neuroendocrine carcinoma (NEC) [7,8].

NEC as a cause of HHM is rare relative to other causes [9]. In one study of 138 patients with PTH-rP-mediated hypercalcemia, NEC was present in only 2.2% of cases [2]. Therefore, other causes of hypercalcemia must be considered when a patient has NEC, including hyperparathyroidism from multiple endocrine neoplasia (MEN) type 1 and local factors secreted by bone tumors [10]. Nonetheless, NEC as a cause for HHM is possible and herein we describe a case of a 31-year-old patient with HHM associated with elevated PTH-rP.

## Case presentation

**Figure 1:**
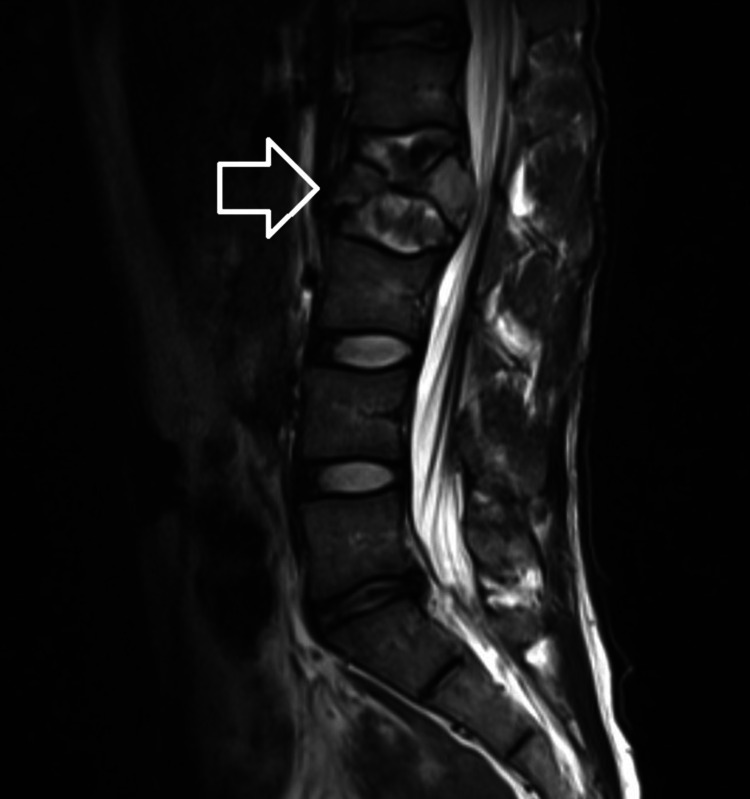
Pathologic fracture of the lumbar spine. Pathologic compression fracture of L2 with retropulsion resulting in moderate spinal canal stenosis and mild right L2/L3 neuroforaminal narrowing.

**Figure 2:**
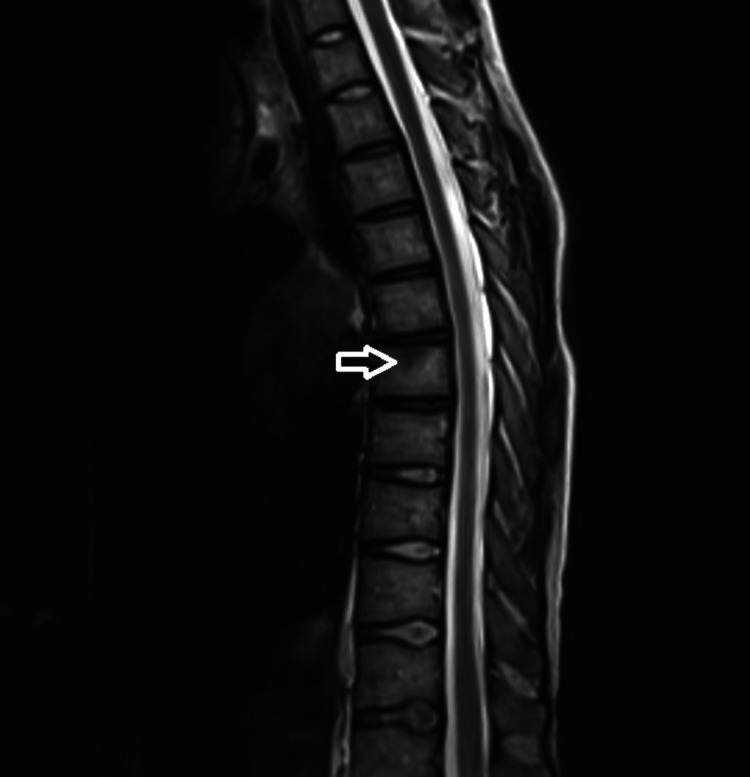
Pathologic fracture of the thoracic spine. Indeterminate small focus of low T1 and high T2/short tau inversion recovery (STIR) signal in the superior endplate of T7.

A 28-year-old male presented to his primary care manager with back pain after falling in the shower. A trial of conservative management was unsuccessful and the pain worsened over the following months. An MRI showed a pathologic fracture of the lumbar spine and an indeterminate lesion to the thoracic spine (Figures 1, 2).

**Figure 3:**
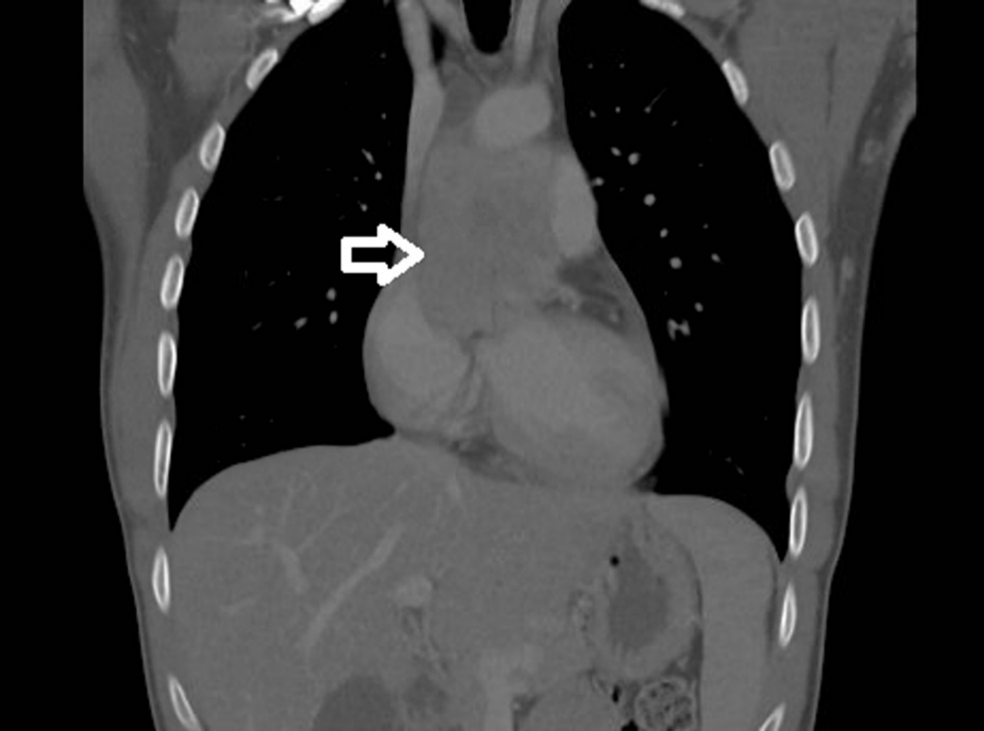
Mass surrounding great vessels of the heart. Infiltrative medial mediastinal mass demonstrating narrowing of the right pulmonary artery with possible invasion. Mass effect present on the left atrium, superior vena cava, and carina. Mass abuts the aortic root/ascending thoracic aorta.

Computed tomography (CT) scan of the chest showed a mediastinal mass encasing many of the great vessels surrounding his heart (Figure 3).

Labs showed hypercalcemia, which was successfully treated with calcitonin and zoledronic acid before becoming complicated by hypocalcemia.

**Figure 4:**
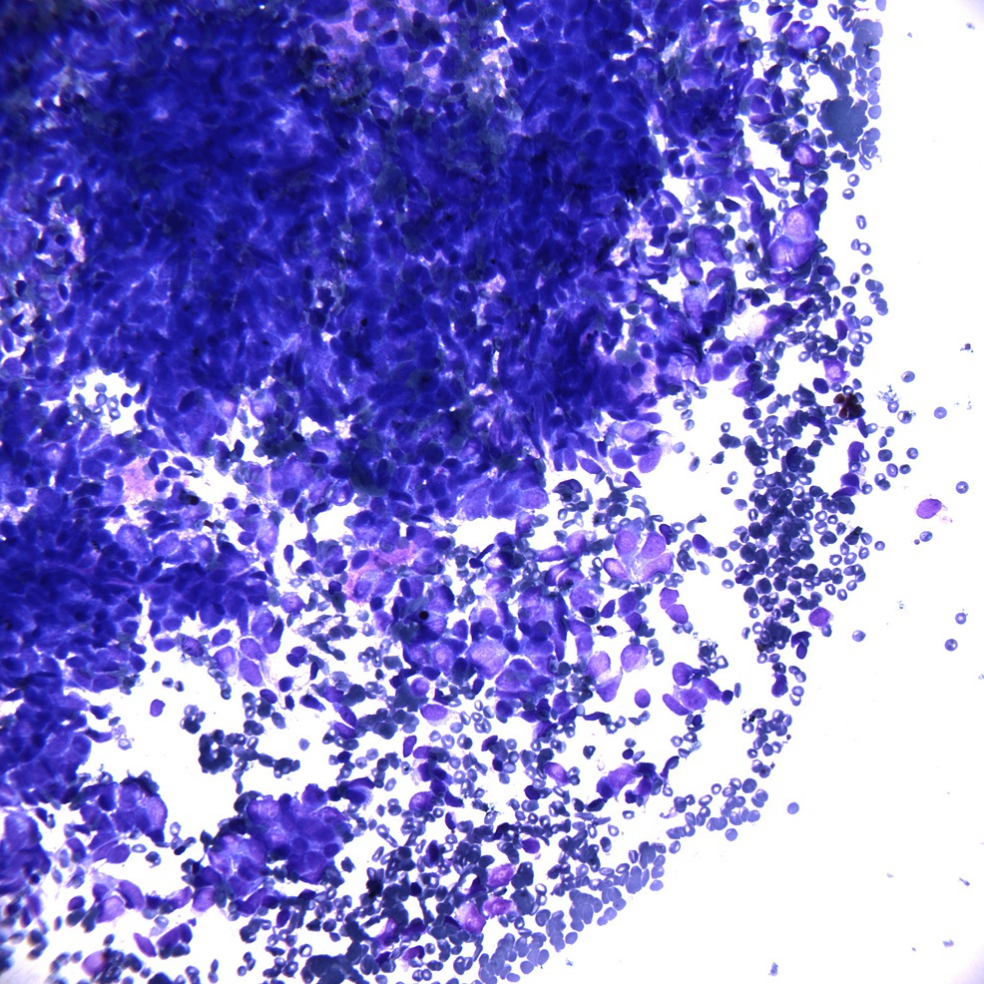
Fine needle aspiration smear stained with Diff-Quik, 40x objective.

**Figure 5:**
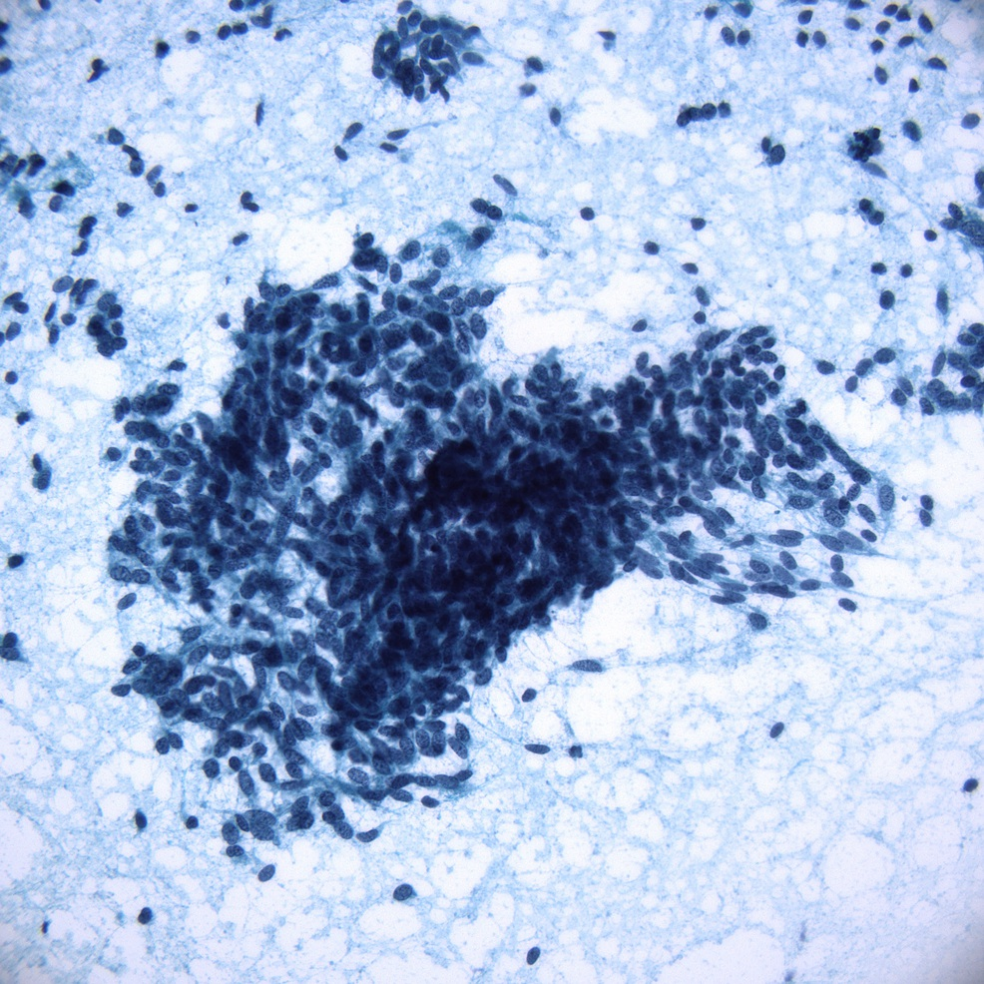
Fine needle aspiration smear stained with Pap stain, 40x objective.

The patient underwent an endobronchial ultrasound bronchoscopy (EBUS) with biopsy, which showed a malignant spindle and epithelioid cell neoplasm (Figures 4, 5).

**Figure 6:**
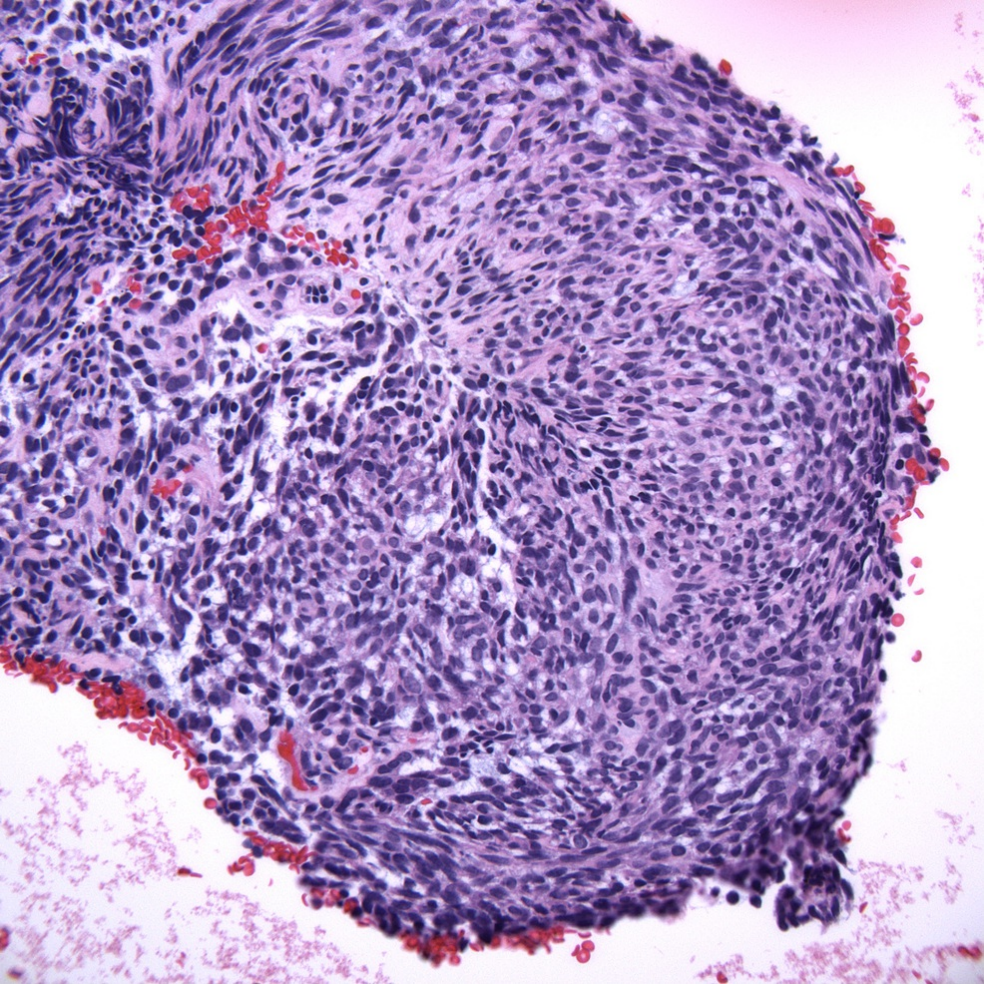
Hematoxylin and eosin-stained slide of the cell block, 40x objective.

**Figure 7:**
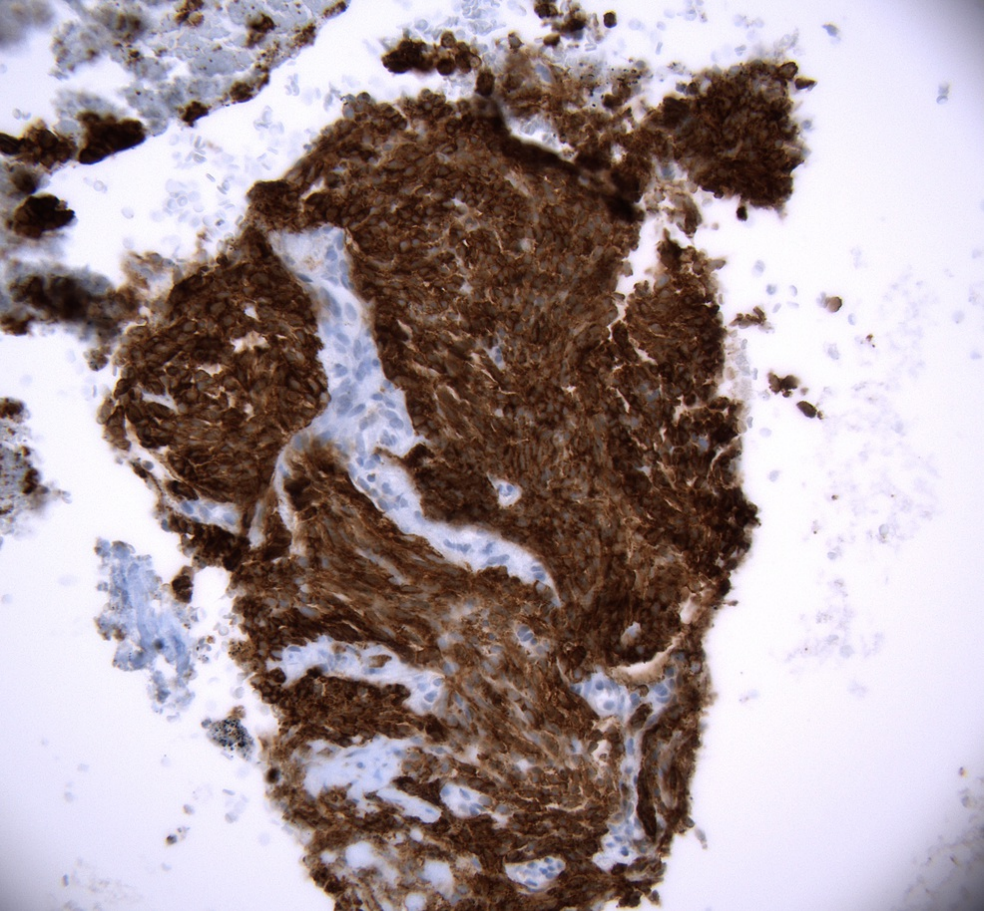
Immunohistochemistry of cell block stained with pan-cytokeratin stain (Lu-5), 40x objective.

**Figure 8:**
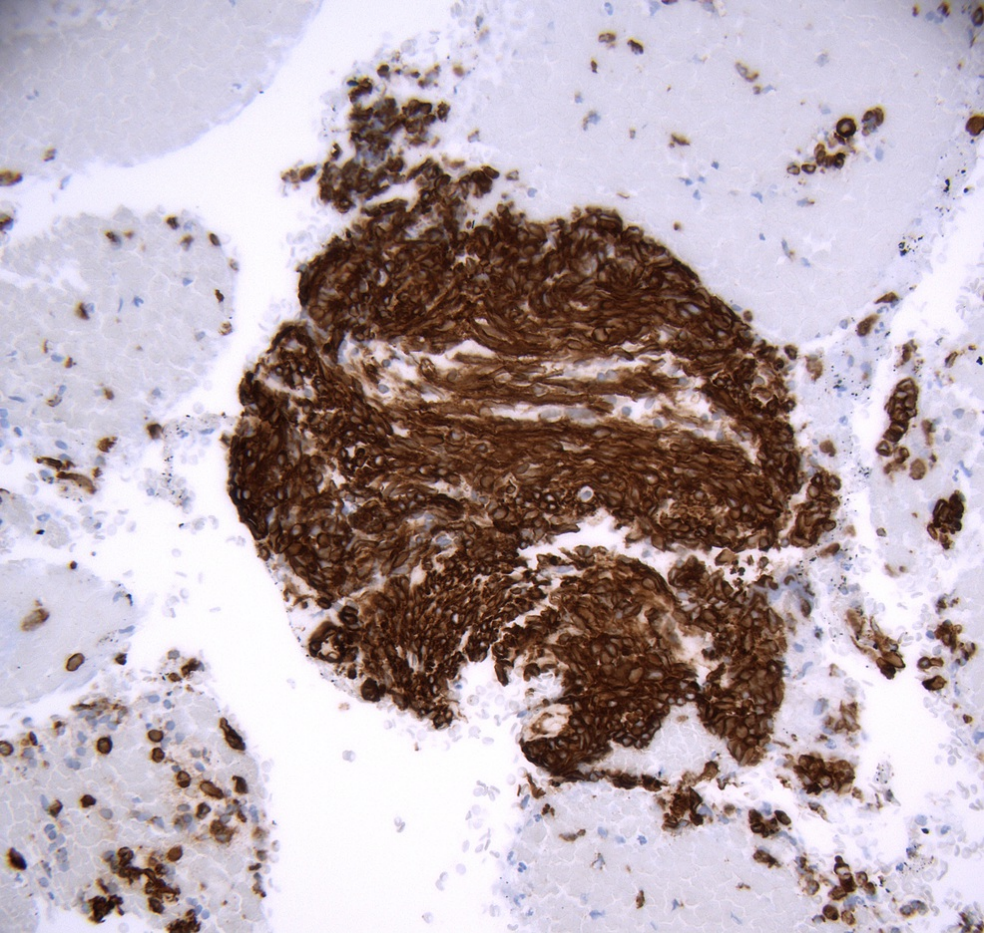
Immunohistochemistry of cell block stained with neuroendocrine marker (CD56), 40x objective.

He then had a core needle biopsy to a lesion that had been identified at L2; this also showed a spindle cell neoplasm, with similar cytomorphology and immunoprofile (Figures 6-8).

Analysis from the first sample showed the tumor cells were diffusely positive for Lu-5, P40, CD56, and vimentin, focally positive for transducin-like enhancer of split-1 (TLE1), and rarely positive for synaptophysin and DOG1. They were negative for S100, thyroid transcription factor 1 (TTF1)/napsin, CD117, CD45, terminal deoxynucleotidyl transferase (TdT), placental alkaline phosphatase (PLAP), Oct4, chromogranin, myogenin, and desmin. The final diagnosis was made after molecular analysis failed to detect an SS18 rearrangement. This finding suggested against the diagnosis of synovial sarcoma, and a diagnosis of high-grade NEC was made based on histologic grounds alone.

The patient was referred for a clinical trial. He was found to have progression of his lumbar fracture, for which he received vertebroplasty at levels of T12-L4 and surgical stabilization and fusion. This was followed by palliative radiation therapy (RT) (4,500 cGy in 15 fractions) to the mediastinum and a metastatic L2 lesion. He also received RT to T12-L4 at 3,000 cGy, followed by cisplatin and etoposide for six cycles. This regimen resulted in stable disease for about eight months, when he was found to have progression of disease with bony involvement. He then received targeted RT, followed by carboplatin, etoposide, and atezolizumab, before starting maintenance therapy with atezolizumab.

Seven months later, he received another course of radiation for a new boney disease complicated by the development of hypercalcemia. Due to previous hypocalcemic events when treated with zoledronic acid and calcitonin, weekly injections of denosumab 120 mg were trialed, without significant effect. After another six months, the patient’s disease again progressed, and he enrolled in a clinical trial assessing a humanized antibody conjugated to monomethyl auristatin E. Shortly thereafter, he presented with a calcium level of 14.6 mg/dL and alkaline phosphatase (ALP) of 399 U/L, treated with normal saline and a fourth weekly dose of denosumab with normalization of his calcium.

Repeat labs four days later showed calcium of 14.6 mg/dL, phosphorus of 1.2 mg/dL, WBC of 17.21 per microliter, and aspartate aminotransferase (AST)/alanine aminotransferase (ALT) of 77/87 IU/L. Physical exam was notable for tachycardia and a known epigastric protrusion secondary to the underlying tumor. Due to his previous hypocalcemic episodes when treated with bisphosphonates and calcitonin, and the potential for disqualification from his clinical trial if he were to receive high dose glucocorticoids, he was treated with intravenous fluids and electrolyte repletion alone, which temporarily resulted in a drop in serum calcium to 11.9 mg/dL. He continued to receive intravenous hydration and aggressive electrolyte repletion, but his calcium nonetheless increased to 15.5 mg/dL. Other notable labs included: PTH-rP (25 pmol/L), 1,25-dihydroxyvitamin D (513 pg/mL), parathyroid hormone (PTH, 6 pg/mL), hemoglobin (10.6 g/dL), WBC (18), lactate dehydrogenase (LDH, 774 U/L), and persistently elevated liver function tests. He was given 90 mg of pamidronate IV and started on 10 mg of prednisone daily, which resulted in a drop of his calcium to 11.5 mg/dL before discharge.

One week after discharge, he presented again with a calcium level of 13.1 mg/dL, increasing to 15.2 less than a month afterward. Thereafter he was unenrolled from the clinical trial due to progressive liver metastases. Calcium remained persistently elevated with PTH-rP (47 pmol/L). The patient reported a desire to continue pursuit of anti-neoplastic therapy; however, he was not qualified for additional chemotherapy due to persistent hypercalcemia. To assist him in achieving this goal, all vitamin D supplementation was ceased and a trial of cinacalcet was initiated.

While initiating cinacalcet, the patient subsequently trialed multiagent cytotoxic chemotherapy; however, due to intractable nausea, therapy was ceased. During this time, his calcium levels continued to range from 9.9 to 16.4 mg/dL, with some improvement at the high end after intravenous fluids. He began palliative treatment with leucovorin, 5-fluorouracil, irinotecan (FOLFIRI) and was nauseous frequently, requiring treatment to be intermittently held. He was then hospitalized again with a calcium level of 16 mg/dL, which improved to 12.2 with intravenous fluids in addition to the patient’s home regimen of denosumab, prednisone, and cinacalcet. Calcium over the next month ranged from 13 to 15.7 mg/dL. ALP ranged from 357 to 511 U/L.

**Figure 9:**
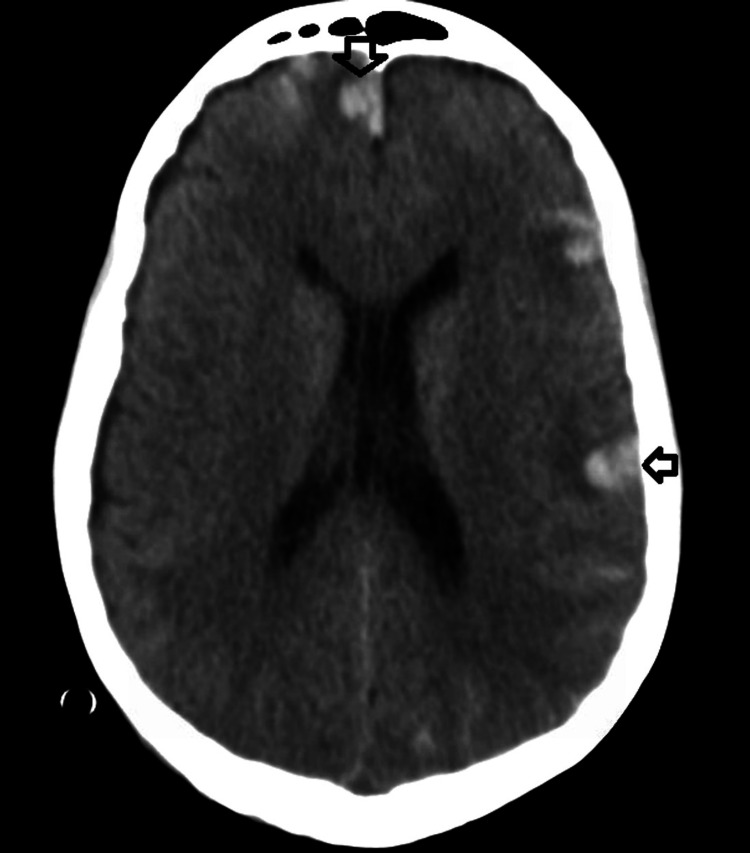
Subarachnoid hemorrhages. Multiple peripheral cortical hemorrhagic contusions with surrounding edema, subarachnoid hemorrhage, and planum sphenoidale extra-axial collection.

The patient was ultimately hospitalized again after being found down. A CT scan showed a diffuse subarachnoid hemorrhage without a midline shift (Figure 9), after which the patient had seizure-like activity.

Presentation labs showed WBC of 28.3, calcium of 15.7 mg/dL, and ALP of 357 U/L. He required intubation and was transferred to the intensive care unit, where he was treated with antiepileptics and antibiotics for ventilator-associated pneumonia. Calcium decreased to 9.9 mg/dL with his home cinacalcet and intravenous fluids. The patient remained confused and somnolent throughout the admission, severely malnourished, and was discharged home on hospice care.

## Discussion

HHM is a paraneoplastic process caused by elevations in PTH-rP and is rarely caused by NEC [9]. This patient’s refractory hypercalcemia despite multiple calcium-reducing agents made treatment difficult, as balancing the proper level of treatment with the risk of precipitating hypocalcemia had to be juggled. Ultimately, cinacalcet was utilized for refractory hypercalcemia, as it has successfully been used in the setting of NEC with HHM before [11].

Furthermore, elevations in vitamin D levels are uncommon in HHM [12]. While most HHMs are associated with normal to low levels of 1,25-dihydroxyvitamin D, our patient had over six times the upper limit of normal when his PTH-rP was first discovered. PTH levels, on the other hand, are usually low, as was the case with this patient.

Ultimately, the most effective treatment for paraneoplastic association HHM is the treatment of the primary disease [13]. In this context, the patient had evidence of disease that was refractory to multiple agents. As such, treatment of hypercalcemia in cases like this proves to be difficult and alternative methods are often employed. Given the patient received a partial response to therapy with FOLFIRI while receiving cinacalcet, this combination of factors may have assisted with improvement in hypercalcemia and facilitated outpatient treatment.

## Conclusions

We report a case of a patient with NEC who had HHM. The patient was treated with multiple calcium reduction agents including IV fluids, denosumab, zoledronic acid, pamidronate, calcitonin, and prednisone, with variable effects. Cinacalcet may be considered an adjunct therapy in cases of refractory hypercalcemia secondary to malignancy, although its efficacy remains unknown in this clinical setting.
